# Large-Area Nanolattice Film with Enhanced Modulus, Hardness, and Energy Dissipation

**DOI:** 10.1038/s41598-017-09521-6

**Published:** 2017-08-22

**Authors:** Abhijeet Bagal, Xu A. Zhang, Rahnuma Shahrin, Erinn C. Dandley, Junjie Zhao, Felipe R. Poblete, Christopher J. Oldham, Yong Zhu, Gregory N. Parsons, Christopher Bobko, Chih-Hao Chang

**Affiliations:** 1 Department of Mechanical and Aerospace Engineering, North Carolina State University, Raleigh, North Carolina 27695 United States; 2 Department of Civil, Construction and Environmental Engineering, North Carolina State University, Raleigh, NC 27695 United States; 3 Department of Chemical and Bimolecular Engineering, North Carolina State University, Raleigh, North Carolina 27695 United States

## Abstract

We present an engineered nanolattice material with enhanced mechanical properties that can be broadly applied as a thin film over large areas. The nanolattice films consist of ordered, three-dimensional architecture with thin-shell tubular elements, resulting in favorable modulus-density scaling (*n* ~ 1.1), enhanced energy dissipation, and extremely large material recoverability for strains up to 20% under normal compressive loading. At 95.6% porosity, the nanolattice film has demonstrated modulus of 1.19 GPa and specific energy dissipation of 325.5 kJ/kg, surpassing previously reported values at similar densities. The largest length scale in the reported nanolattice is the 500 nm unit-cell lattice constant, allowing the film to behave more like a continuum material and be visually unobservable. Fabricated using three-dimensional colloidal nanolithography and atomic layer deposition, the process can be scaled for large-area patterning. The proposed nanolattice film can find applications as a robust multifunctional insulating film that can be applied in integrated photonic elements, optoelectronic devices, and microcircuit chips.

## Introduction

Naturally occurring cellular materials such as biological exoskeletons^1–4^, bones^5^, cork, and wood^6^ are lightweight and simultaneously exhibit superior mechanical properties. In these materials, the composition and architectural arrangement of constituent elements at the micro/nano-scale result in improved stiffness modulus, strength, and energy absorption. Inspired by biology, engineered cellular materials such as metal foams^7, 8^, polymer foams^9–11^, and aerogels^12–16^ have been demonstrated by researchers. Such cellular materials can find applications in thermal insulation^11, 15, 16^, acoustic damping^15, 16^, shock absorption^17, 18^, as well as in water purification and catalytic reactions^16^ due to increased surface area. Similar to the biological examples, many of these materials have micro/nano-scale geometrical constituent elements that are randomly oriented, which are the result of the stochastic nature of the synthesis and fabrication processes involved. Therefore, while such materials can have extremely low density, it can be difficult to precisely control the architectural arrangements. It is well known that the mechanical properties of randomly porous cellular material degrade rapidly with lower density. For example, the elastic modulus for cellular solids can be related to density through the power-law relationship $E/{E}_{s}\,\alpha \,{{\rho }_{R}}^{n}$, where ${\rho }_{R}$ is relative density, *n* is scaling factor, and *E*_*s*_ and *E* are elastic moduli for solid and cellular materials, respectively. For random architectural arrangements the modulus and yield strength scale with a power of *n* = 3–4^5, 19–22^. As a result, materials with random architectures are mechanically less durable at low densities.

Recent research in lightweight structures and materials has focused on mitigating the mechanical degradation for more favorable density scaling. It has been demonstrated that by having an ordered arrangement of constituent elements, mechanical properties of a porous material can be significantly improved to achieve better density scaling^20–27^. Such ordered nano/microlattices have resulted in mechanically robust structures with modulus and yield strength scaling of *n* = 1–2. The constituent elements can also be made hollow to achieve ultra-low density, while maintaining similar enhanced scaling. In addition, unique architectural arrangements have shown remarkable material properties such as energy dissipation^23, 28–30^, negative Poisson’s ratio^31^, ductile-like recoverability^22^, and high stretchability^32^. Through deterministic design of the micro/nanolattice architectures, the material response can be better tailored to achieve optimal mechanical response.

While these research present a significant breakthrough, there are some inherent limitations in the current fabrication approaches. Recent work demonstrating enhanced density scaling of mechanical properties are based on micro-stereolithography^21^ and two-photon lithography^22, 33^, and such techniques have limited spatial resolution and patterning throughput, respectively. Therefore, while the demonstrated results have nanoscale elements, the lattice is generally larger and ranges from 1–200 μm^21, 22, 24, 33, 34^. Such materials resemble more like a “structure” rather than a continuous “material” as a result of the relatively large lattice constant, and can be difficult to integrate for many applications. In addition, while two-photon lithography is an attractive process due to the ability to create arbitrary 3D patterns, it relies on serial direct-write patterning and can be difficult to apply over a large area in a cost-effective manner. Therefore, while recent works have demonstrated exciting novel nanomechanics, the nanolattices coverage area are generally limited to 100 × 100 μm^2^ range and implementation over large area remains a significant challenge.

Here we demonstrate an ordered 3D nanolattice film with enhanced mechanical properties for normal compressive loading using scalable nanofabrication techniques. This nanolattice film is enabled by 3D colloidal nanolithography, which can be scaled to pattern nanolattice structures over macroscale areas. With lattice constants at least an order of magnitude smaller than most existing work, this nanolattice behaves as a continuum “material” as opposed to a “structure” and can be widely applied as thin film over any substrate. Having near sub-wavelength lattice constant, the nanolattice film cannot be observed visually and allows facile integration into a broad range of surfaces and material systems. This 3D nanolattice film has also demonstrated enhanced mechanical behavior including near-linear modulus scaling with density, and among the highest specific energy dissipation capacity reported for porous materials. The proposed lightweight nanolattice film also exhibits near-complete material recovery for the thinnest shells (*t* < 10 nm). In addition to novel mechanical properties, the nanolattice film has demonstrated refractive index very close to air^35^. Having over 95% porosity, such highly porous material can also have dielectric constant and thermal conductivity very close to air, which is often observed in aerogels. Being simultaneously mechanically robust and optically, electrically, and thermally behaving like air, the nanolattice film can find a potential application as a multifunctional insulating film that can be integrated into multilayer optoelectronic devices, photonic elements, and microcircuits.

## Results and Discussion

The fabrication approach is based on the patterning of a periodic 3D photoresist template using colloidal phase lithography^36^ and atomic layer deposition (ALD), and has been reported previously^35^. The pattern size of this technique depends solely on the area of the assembled monodispersed particles, and full-wafer assemblies have been demonstrated^37^. While local assembly imperfection, such as point defects and grain boundaries, can exist, the defect area density is generally small (<5%). Samples fabricated for this work were typically ~3 × 3 cm^2^. Requiring only a single flood illumination, the exposure time of the 3D lithographic process is on the scale of minutes. The lattice constant of the resist template depends on the particle diameter, which is typically selected to be around 200–700 nm. While the current work focuses on film with around 1 μm thickness, this fabrication technique has demonstrated thicker films around 5–10 μm^38^. This photoresist template can then be conformally coated with thin films of various material compositions using ALD. In this work, Al_2_O_3_ and ZnO were selected to fabricate the nanolattice films due to their attractive bulk properties. The fabrication details used in the experiment is described in Supplementary Information A.

It is well-understood that bending of elements in cellular materials show higher modulus scaling (*n* ~ 2), resulting in weaker structures at low density. In contrast, the mechanical properties can be enhanced by aligning the constituent elements along the loading direction, making the elements stretch and compress to allow the scaling to be closer to the theoretical limit (*n* = 1)^18, 20^. To achieve near-linear scaling to compressive and tensile loads normal to the surface, we have designed the nanolattice film to have constituent elements that are vertically aligned and interconnected. This can be achieved lithographically by using a low exposure wavelength (*λ* = 325 nm) to particle diameter (*D* = 500 nm) ratio, which governs the unit-cell geometry^38^. After removing the sacrificial polymer template, the resulting structure has a unique arrangement of tubular thin-shell elements, which imparts enhanced mechanical stability to the nanolattice film under normal compressive loading. This is analogous to micron-scale structural anisotropy obtained through processing in Damascus steel to achieve better properties^39^. Note that such an architecture is effective only when the force is normal to the film, and may be weaker along other directions. This lithography configuration was used to fabricate all the samples.

**Figure 1:**
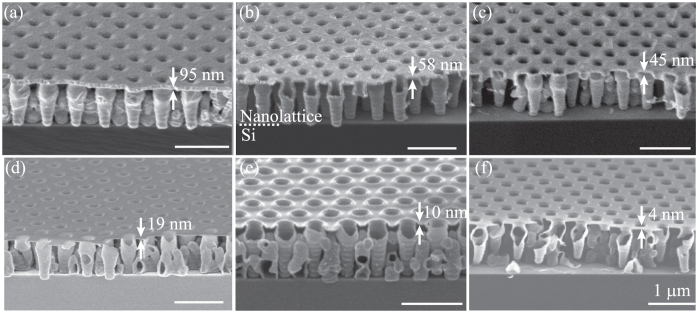
Scanning electron micrographs of samples used for mechanical testing. (**a–c**) Cross-sectional image of ZnO nanolattices with thicknesses 95 nm, 58 nm, and 45 nm respectively. (**d–f**) Cross-sectional SEM image of Al_2_O_3_ nanolattices with thicknesses 19 nm, 10 nm, and 4 nm respectively. The thinnest nanolattice tested for mechanical properties had a shell thickness of 4 nm. Cross-sectional image of 4 nm nanolattice shows the structure is free-standing and does not collapse during template removal process.

Cross-sectional scanning electron microscope (SEM) images of fabricated 3D nanolattice films are shown in Fig. 1. ZnO nanolattices were fabricated with thicker layers than Al_2_O_3_ nanolattices; 95, 45, and 30 nm-thick samples are shown in Fig. 1a–c. ZnO nanolattices with thinner shells were difficult to fabricate, as the polycrystalline ZnO film becomes porous and collapses during the photoresist template removal process (Supplementary Information B). Al_2_O_3_ nanolattice films can be fabricated with thinner shells; 19, 10, and 4 nm-thick samples are shown Fig. 1d–f. The thinnest Al_2_O_3_ sample tested for mechanical properties had a shell thickness of 4 nm. Compared to ZnO, Al_2_O_3_ is amorphous, mechanically more resilient and can withstand the template removal process with a thinner shell thickness. As seen from the cross-sectional SEM images, the architectural arrangement mainly consists of vertically aligned thin-shell tubular column with secondary interconnecting structures in between. This unique ordered arrangement of thin-shell tubular columns results in enhanced mechanical properties under compressive loading. For all the samples tested in this study, the lithography parameters were kept constant and the porosity was changed by varying the thickness of the ALD layer. Nanolattice films with porosities ranging from 60.9–95.6% and 27.2–65.6% for Al_2_O_3_ and ZnO, respectively, were tested for mechanical properties.

Mechanical properties of both Al_2_O_3_ and ZnO nanolattice films were tested by applying uniaxial compressive loading using nanoindentation. In these experiments a spherical diamond indenter with 10 µm radius was applied with a predefined load on the nanolattice film. The indenter had a contact area spanning over a few unit cells, which ensures that the applied load predominantly acts normal to the sample surface and the nanolattice film behaves like a continuum material. To avoid the substrate effect on the measurement of mechanical properties, the maximum nanoindentation depth *h* was kept close to 10% of the nanolattice film thickness, which is typically around ~1 μm. Al_2_O_3_ and ZnO nanolattice films with varying shell thicknesses were compared for their mechanical properties by calculating the effective indentation modulus from the slope of unloading curve using the Oliver-Pharr method^40^.

**Figure 2:**
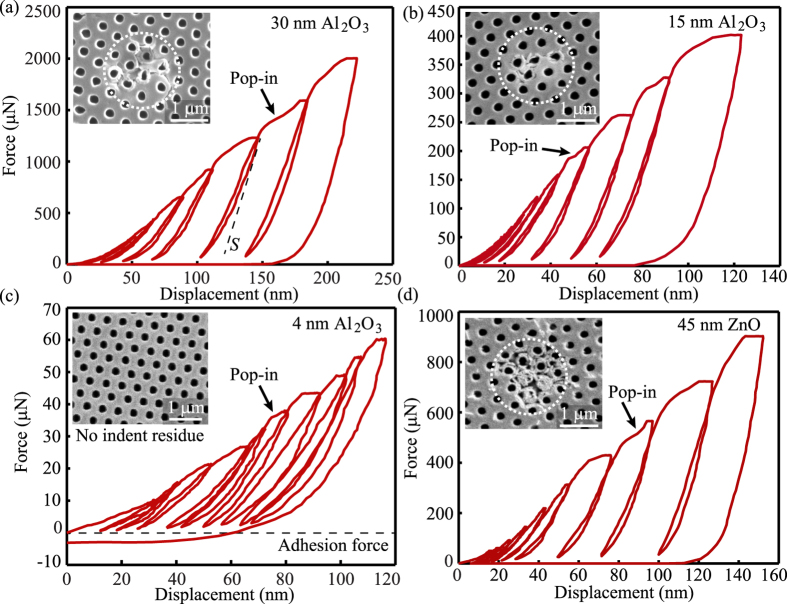
Mechanical testing of nanolattice using nanoindentation. (**a**) Typical cyclic load-displacement curve for 30 nm Al_2_O_3_ nanolattice. ‘Pop-in’ indicates first instance of mechanical failure of nanolattice film. Inset shows post-indent SEM image with residual indentation imprint, showing brittle fracture of top planar layer. (**b)** Load-displacement curve for 15 nm Al_2_O_3_ nanolattice showing similar loading-unloading behavior as 30 nm Al_2_O_3_ nanolattice. This is the thinnest sample tested which failed by fracture of top planar layer. (**c**) Load-displacement curve for 4 nm Al_2_O_3_ nanolattice showing gradual ‘pop-in’ event. Inset shows post-indent SEM with no residual indentation imprint. Non-zero adhesion force from van der Waal’s attraction between the diamond indent and Al_2_O_3_ nanolattice indicate near-complete recovery post indentation. (**d**) Load-displacement curve for 45 nm ZnO nanolattice showing ‘pop-in’ event similar to thicker Al_2_O_3_ nanolattice. Inset shows post indent SEM image with brittle fracture around holes.

For the nanolattice films tested in this study, a cyclic incremental loading scheme was selected for nanoindentation characterization (Supplementary Information C). This approach helps to determine indentation modulus prior to ‘pop-in’ events, which signify the first instance of mechanical failure due to structural collapse either by yielding, buckling, or fracture. A representative nanoindentation incremental cyclic loading-unloading curve for a 30 nm Al_2_O_3_ nanolattice with a ‘pop-in’ event noted is shown in Fig. 2a. After the structure collapse at the ‘pop-in’ event, the nanolattice densifies and the architectural arrangement of the structure changes, which will affect the mechanical properties of the structure during subsequent incremental loading cycles. To avoid this effect, only the cycles before the ‘pop-in’ event were considered for calculating modulus, hardness, and the energy dissipation. Note this typically occurs when the indentation depth is less than 10% of the film thickness, therefore eliminating any substrate effects. To get reliable data for mechanical properties, each nanolattice sample was tested at multiple locations.

After testing, top-view SEM images were taken for the indented locations to ensure that the collected data was from a defect-free location (supplementary Information C), and only such data was considered for further calculation of mechanical properties. The SEM image in Fig. 2a shows the top-view residual indentation on a 30 nm Al_2_O_3_ nanolattice sample. The image shows that the sample failed under compressive loading with cracks appearing around the holes. Cross-sectional images of the residual indent, which might reveal additional failure modes like buckling or rupture of primary tubular shell with increased load after the ‘pop-in’ event, were difficult to obtain since sample cleaving would result in further lattice damage. Similar failure behavior was observed for both Al_2_O_3_ and ZnO nanolattice films with shell thickness larger than 10 nm. Figure 2b,d show the load-displacement curves and post indent SEM images for 15 nm Al_2_O_3_ and 45 nm ZnO nanolattice films, respectively. Nanolattice films with shell thickness smaller than 10 nm had serrated load-displacement curves as shown in Fig. 2c, exhibiting ductile-like behavior. The post indent SEM images do not show fracture on top of the planar layer and residual indentation imprints.

**Figure 3:**
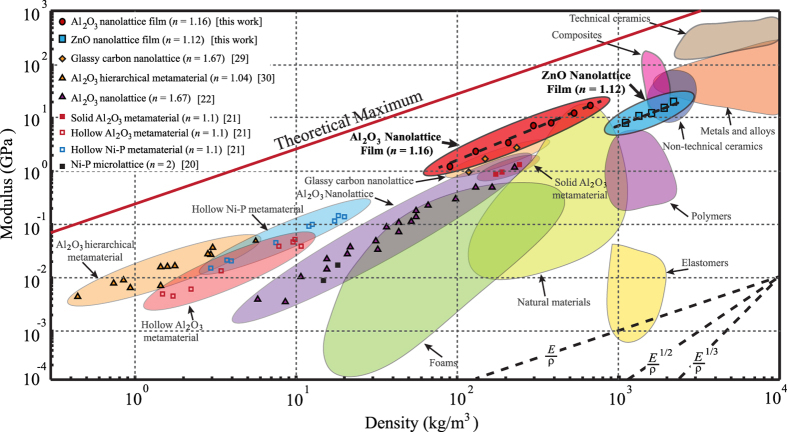
Modulus-density Ashby Chart. The indentation moduli of Al_2_O_3_ and ZnO nanolattice are plotted against density for all the tested samples on an Ashby chart. The results for both ALD materials follow power law with scaling *n* ~ 1.1.

The nanoindentation data were analyzed to calculate the mechanical properties of nanolattice films (Supplementary Information D). The compressive indentation modulus was calculated by averaging the slope of unloading curves for incremental loading-unloading cycles before the ‘pop-in’ event. This approach reduced the effect of over predicting of modulus due to densification of nanolattice, and mitigates initial contact error during first loading-unloading cycle. The indentation modulus (*E*) of all nanolattices was plotted against the density (*ρ*) in Fig. 3, and a near-linear scaling of 1.16 and 1.12 between modulus and density were observed for Al_2_O_3_ and ZnO nanolattices, respectively. This scaling outperforms^21, 22^ and is comparable^34^ to previously reported micro/nanolattices with larger lattice parameter >10 μm, demonstrating that enhanced modulus scaling can also be achieved at the sub-micron lattice regime. The near-linear modulus scaling was the result of the unique nanolattice geometry which has primary thin-shell tubular columns aligned normal to the surface. The thinnest nanolattice film, constructed from 4 nm-thick Al_2_O_3_ with 95.6% porosity, has demonstrated a modulus of 1.19 GPa at density of 127.6 kg/m^3^. This is higher than the previously reported value of ~0.5 GPa in literature at similar densities for ordered Al_2_O_3_ microlattice^21, 22^. Furthermore, the modulus achieved for 15 nm thick Al_2_O_3_ nanolattice film (*E* = 6.98 GPa) would require twice the volume fraction in porous Al_2_O_3_ aerogel with random architecture^41^.

**Figure 4:**
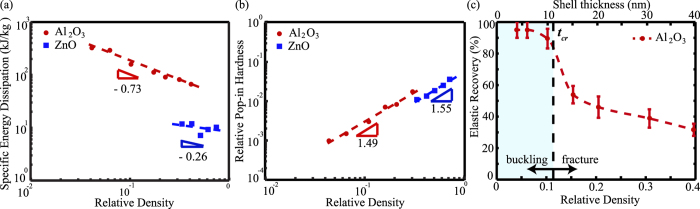
Mechanical Properties of nanolattice film. (**a**) Specific energy dissipation for Al_2_O_3_ and ZnO nanolattice plotted against relative density. The Al_2_O_3_ nanolattice shows more favorable power law scaling. (**b**) Pop-in hardness versus relative density. The nanolattice shows similar hardness scaling for both Al_2_O_3_ and ZnO. (**c**) Elastic recovery of Al_2_O_3_ nanolattice film plotted as a function of relative density and nanolattice shell thickness. Error bars represent standard deviation in elastic recovery.

The non-linear nanoindentation response in the later loading-unloading cycles results from elastic-plastic deformation of the nanolattice film. During unloading, the elastic work done is released by partial recovery of the nanolattice, but the plastic work done will be dissipated through localized, permanent deformations of the structure. Figure 4a plots the dissipated energy per weight by Al_2_O_3_ and ZnO nanolattices as a function of relative density before the ‘pop-in’ event. As expected, the thicker structures dissipate higher total energy before collapse. Specific energy dissipation, calculated by normalizing the total energy by the weight of the solid phase, can be examined to evaluate effectiveness for light-weight applications. For the 4 nm Al_2_O_3_ nanolattice sample, the specific energy dissipation was 325.5 kJ/kg at average indentation lower than 10% (Supplementary Information D), and the total energy absorbed was 1.16 pJ (Table S3). This specific energy dissipation is much larger than the previously reported polymer foams (0.15 kJ/kg at 21 kg/m^3^ density), ordered polymer nanoframes (7.67 kJ/kg at 600 kg/m^3^ density)^18, 23^, and same order of magnitude as carbon-epoxy composite nanostructures (720 kJ/kg at 2000 kg/m^3^)^27^. In addition, the enhanced energy dissipation by the nanolattice film was also achieved at comparatively smaller total strain and density. The energy dissipation capacity ranged from 67.2 to 325.5 kJ/kg and 7.54 to 12.07 kJ/kg for Al_2_O_3_ and ZnO nanolattices, respectively.

The energy dissipation mechanism can be attributed to brittle fracture of the thin-shell elements within the nanolattice material, which dissipates energy through the creation of micro-cracks (and thus new fracture surfaces). Under such a mechanism the high surface-area-to-volume ratio plays a critical role, resulting in higher specific energy dissipation for thinner nanolattices. For the thinnest samples with 4 and 6 nm, the material may also exhibit ductile-like properties and contribute to energy absorption. It is important to note that while the specific energy dissipation is high, the total energy dissipated is in the 1–40 pJ range and is small. Taller nanolattice films are needed to increase the total energy dissipation, and film height of ~10 μm is possible through the proposed lithography process and can be potentially improved further^38^. Metallic materials with high ductility can also be used in place of metal oxides to construct the thin-shell nanolattice, which can further enhance energy dissipation through plastic deformation.

In addition to improved modulus and specific energy dissipation, the nanolattice film also exhibits favorable hardness scaling. The nanolattice hardness was calculated by dividing the load at the ‘pop-in’ event by the indenter contact area at that instance. For the highest porosity sample, the 4 nm Al_2_O_3_ nanolattice had an indentation hardness of 7.8 MPa. The hardness scaling exponents for Al_2_O_3_ and ZnO are 1.49 and 1.55 respectively, as shown in Fig. 4b. Similar scaling exponents for both Al_2_O_3_ and ZnO are the result of the unique architectural arrangement of the nanolattice rather than the ALD material property.

Failure mode analysis of a simplified tubular shell element revealed that the two failure modes, fracture and buckling, are governed by critical thickness *t*_*cr*_ (Supplementary Information E). The nanolattice undergoes a fracture-dominated failure mode for film thickness *t* > *t*_*cr*_ where the top planar layer cracks before buckling of tubular columns. For *t* < *t*_*cr*_, the initial failure in the nanolattice occurs by buckling of tubular columns, which allows for the film to recover after removing the load. The buckling can be observed in the FEA simulation shown in Supplementary Information D. The critical thickness is a function of ALD material properties and tubular column geometry; it was calculated to be 11.5 nm for Al_2_O_3_ (Supplementary Information E). This prediction, first reported for nanolattice with larger lattice parameter of 5 μm^22^, is supported by the nanoindentation results. First, thicker nanolattices governed by fracture-dominated failure mode have smooth loading-unloading curves but a sharp ‘pop-in’ event, indicating brittle failure of the top planar film. For thinner nanolattices the loading-unloading curve was serrated and the ‘pop-in’ event was gradual and less pronounced, indicating buckling behavior. Note that fracture can still occur locally within the thinner shell nanolattices to dissipate energy (indicated in Fig. 4a), since the actual sample geometry is more complex than the simplified analytical shell model. Second, all Al_2_O_3_ samples with shell thickness *t* < *t*_*cr*_ had a negative force of indentation when being completely unloaded, as shown for 4 nm Al_2_O_3_ nanolattice film in Fig. 2c. This is a result of adhesion force due to van der Waal’s attraction between the diamond indenter and the recovered nanolattice film (Supplementary Information F). The non-zero adhesion force, even when the nanolattice had regained its original height, indicates that the nanolattice had fully recovered from compressive loading during indentation. This adhesion force can also be observed for samples with *t* > *t*_*cr*_ during unloading, but is removed after the tip loses contact with the non-recovered film. Third, the planar top film can be examined post-indent using top-view SEM images to examine indentation footprint.

The recoverability of Al_2_O_3_ nanolattice films after complete unloading of the samples is estimated from the indentation plots, and is plotted as a function of relative density in Fig. 4c. Samples with *t* > *t*_*cr*_ failed by fracture of the top planar layer and show only partial recovery. Whereas, samples with shell thickness *t* < *t*_*cr*_ show ductile-like behavior, resulting in large recovery after unloading. As predicted by failure mode analysis, thinner nanolattices fail by shell buckling of a primary tubular column and do not result in systematic collapse. The 4 nm and 6 nm Al_2_O_3_ nanolattices show near-complete recovery from compressive strains of about 20%, which corresponds to a total indentation depth of 120 to 180 nm. This can be confirmed from post indent SEM images (Fig. 2c and Supplementary Information C), which do not display any post indentation residual imprint on the top surface. Note localized structural damage under the top layer is anticipated and warrants future studies, but the observed damage for *t* < *t*_*cr*_ is not catastrophic and therefore does not deter nanolattice film recovery.

The failure mechanism for nanolattice films depends on ALD material properties, shell thickness and diameter of the tubular column. For all the nanolattice films tested in this work, the outer diameter of the tubular column was maintained constant to be 252 nm, which resulted in *t*_*cr*_ = 11.5 nm for Al_2_O_3_ nanolattice films. If the diameter of the tubular column was reduced further by making lattice constants smaller, *t*_*cr*_ would occur at lower shell thickness. Such arrangement will result in stiffer nanolattice films as the structure will be more difficult to fail by shell buckling. Larger tubular column diameters allow *t*_*cr*_ to occur at higher shell thickness, resulting in easier shell buckling under compressive loading. Such structures are excellent for making recoverable material, but are inherently weaker since they fail at lower stress.

**Figure 5:**
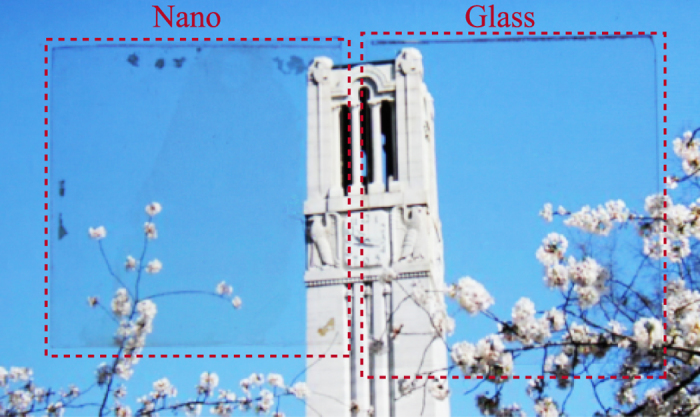
Picture of 10 nm Al_2_O_3_ nanolattice sample and bare glass. “Nano” is the Al_2_O_3_ nanolattice film with 10 nm shell thickness. A display screen was used as backlit background. Scale bar is 1 cm.

The robust nanolattice films fabricated in this work have uniform lattice period of 500 nm over a large area and cannot be readily observed as they visually resemble a clear film coating. A 10 nm-thick Al_2_O_3_ nanolattice film fabricated on glass (“Nano”) illustrates broadband optical clarity that is similar to the adjacent bare glass slide (“Glass”), as shown in Fig. 5. Note the two samples appear indistinguishable under visual observation. As the robust transparent nanolattice material can be applied as a thin film over large areas, it can potentially be integrated into multilayer photonic elements to act as an insulating low-index layer. To improve clarity further, a nanolattice with smaller lattice constants in the 200 nm range is needed and is currently under study. However, this will have to be accompanied by further thickness reduction in order to achieve the same density. The absolute modulus of the nanolattice film also can be improved further by having more close-packed arrangement of tubular columns.

The high specific energy dissipation of the nanolattice film could be useful in ultra-lightweight energy absorption applications where the load is normal to the surface. The vertically aligned, interconnected nanolattice material can also be useful in applications involving unidirectional tensile loading. Future work will focus on using thicker films to enhance total energy absorption. Though the vertically aligned geometry resulted in superior performance for normal loads, this architectural arrangement of nanolattice may be weaker for loading in the direction in the plane of the film. Such anisotropic response can be examined by using indenter tip with radius of curvature smaller than the lattice constant. Currently mechanical tests involving shear loading of the nanolattice film to understand the effect of material anisotropy is underway. The effect of structure symmetry on Poisson’s ratio of the nanolattice film is also of interest and will be analyzed in future work.

## Conclusion

In summary, we have demonstrated a new class of mechanically robust 3D nanolattice film which can be widely applied over any surfaces as a thin film. Under normal compressive loading, the nanolattice film demonstrates near-linear scaling between relative modulus and density, high specific energy dissipation, and near-complete recoverability from strains of about 20% for the thinnest shell samples. The lattice spacing of 500 nm is the smallest demonstrated to date for nanolattices, and as a result behaves more as a continuum material and is not visually observable. Using both colloidal 3D nanolithography and ALD, the fabrication process allows precise control over relative density of the nanolattice and ALD material composition. The highly porous nanolattice film also have refractive index close to air, and can potentially be used as a multifunctional insulating film that can be applied in integrated photonic elements, optoelectronic devices, and microcircuit chips.

## Methods

### Nanolattice thin-film fabrication

For all the samples, the silicon substrates were spin-coated with 98 nm of antireflection coating (ARC i-CON-16, Brewer Science, Inc.) to reduce back reflection, and a positive-tone photoresist (Sumitomo PFi88A7) of ~1 μm thickness. A close-packed monolayer of polystyrene nanospheres with diameter of 500 nm (2.5% Polybead Nonfunctionalized Microspheres, Polysciences, Inc.) was assembled on the resist and exposure by HeCd laser (*λ* = 325 nm) to pattern the sacrificial photoresist template. Photoresist 3D templates were subsequently coated with ZnO or Al_2_O_3_ using ALD in a custom made viscous-flow, hot walled, vacuum reactor described previously^42–44^. ALD consists of two, self-limiting half reactions that produce a conformal thin film on samples of interest. The reactor was purged with high purity nitrogen gas that was further purified with an Entegris GateKeeper. Trimethyl aluminum (TMA, Strem Chemicals, min 98% pure) was used as a precursor and co-reacted with deionized water to deposit Al_2_O_3_. The ALD recipe used for these experiments was 1/30/1/60 (TMA dose/nitrogen purge/water dose/nitrogen purge, in seconds) with a typical growth rate of 1.1 Å per cycle. The reactor was kept around 550 mTorr at 90 °C. Diethyl zinc (DEZ, Strem Chemicals, min 98% pure) was used as the metal-containing precursor for ZnO. DEZ/Water ALD cycles were used for ZnO deposition with growth rate of about 1.6 Å per cycle. In both cases, silicon substrates were placed in the front and back of the reactor chamber as monitors for film growth and film thickness was determined using an α-SE ellipsometer (J. A. Woollam Co., Inc). The polymer template was removed by combinations of thermal treatment using a furnace (Vulcan 3–130, 100–120 V, DENTSPLY) and/or solvent dissolution using N -Methyl-2-pyrrolidone (NMP). For ZnO samples, the sacrificial photoresist layer was removed by using thermal treatment alone. For thermal treatments, samples were placed in the furnace at room temperature and the temperature was increased to 550 °C, with a ramp rate of 0.3 °C min^−1^. The temperature was maintained at 550 °C for 30 min to ensure that both the photoresist template and the underlying ARC layer were burned off. For Al_2_O_3_ nanolattices the photoresist template was first removed by solvent dissolution method. The samples were placed in NMP for 48 hours to dissolve the photoresist template. Later, the ARC layer was removed using the thermal treatment described earlier.

### Mechanical testing using nanoindentation

Mechanical testing of nanolattice film was carried out by using a CSM Ultra Nanoindentation Tester (UNHT). A spherical diamond nanoindenter with 20 μm diameter was used for indentation. Initially larger indents with 10000 μN load were made to mark two boundaries of the rectangular indentation region as reference marks. These two boundaries, 140 μm apart, contained five indents each, marked with 30 μm distance between adjacent indents. Mechanical properties were measured at smaller load using a cyclic incremental loading approach within the marked area. These smaller indents were made at a predefined distance from the larger indents. This enables identifying the indentation region for post-indent SEM imaging to ensure data considered for the calculation of mechanical properties was from a defect-free region. For each sample the maximum load applied, initial contact load and loading rate were kept constant across all indents. The maximum load was selected such that it would result in at least one ‘pop-in’ event during loading.

## Electronic supplementary material

Supplementary Information

Alumina Nanolattice 10 nm

Alumina Nanolattice 40 nm

## References

[1] Meyers MA, Chen P-Y, Lopez MI, Seki Y, Lin AYM (2011). Biological materials: A materials science approach. J. Mech. Behav. Biomed. Mater..

[2] Meyers MA, Chen P-Y, Lin AY-M, Seki Y (2008). Biological materials: Structure and mechanical properties. Prog. Mater. Sci..

[3] Chen P-Y, Lin AY-M, McKittrick J, Meyers MA (2008). Structure and mechanical properties of crab exoskeletons. Acta Biomater..

[4] Chen P-Y (2008). Structure and mechanical properties of selected biological materials. J. Mech. Behav. Biomed. Mater..

[5] Gibson LJ (2005). Biomechanics of cellular solids. J. Biomech..

[6] Ando K, Onda H (1999). Mechanism for deformation of wood as a honeycomb structure II: First buckling mechanism of cell walls under radial compression using the generalized cell model. J. Wood Sci..

[7] Tappan BC (2006). Ultralow-Density Nanostructured Metal Foams:  Combustion Synthesis, Morphology, and Composition. J. Am. Chem. Soc..

[8] Verdooren A, Chan HM, Grenestedt JL, Harmer MP, Caram HS (2006). Fabrication of Low-Density Ferrous Metallic Foams by Reduction of Chemically Bonded Ceramic Foams. J. Am. Ceram. Soc..

[9] Chanda, M. & Roy, S. K. *Plastics Technology Handbook*, *Fourth Edition*. (CRC Press, 2006).

[10] Lee LJ (2005). Polymer nanocomposite foams. Compos. Sci. Technol..

[11] Liu S, Duvigneau J, Vancso GJ (2015). Nanocellular polymer foams as promising high performance thermal insulation materials. Eur. Polym. J..

[12] Tillotson TM, Hrubesh LW (1992). Transparent ultralow-density silica aerogels prepared by a two-step sol-gel process. J. Non-Cryst. Solids.

[13] Zou J (2010). Ultralight Multiwalled Carbon Nanotube Aerogel. ACS Nano.

[14] Nyström, G., Fernández-Ronco, M. P., Bolisetty, S., Mazzotti, M. & Mezzenga, R. Amyloid Templated Gold Aerogels. *Adv. Mater*. n/a–n/a doi:10.1002/adma.201503465 (2015). 10.1002/adma.201503465 26592185

[15] Hüsing N, Schubert U (1998). Aerogels—Airy Materials: Chemistry, Structure, and Properties. Angew. Chem. Int. Ed..

[16] Pierre AC, Pajonk GM (2002). Chemistry of Aerogels and Their Applications. Chem. Rev..

[17] *Materials Selection in Mechanical Design, Fourth Edition*. (Butterworth-Heinemann, 2010).

[18] *Cellular Solids: Structure and Properties*. (Cambridge University Press, 1999).

[19] Ma H-S, Prévost J-H, Jullien R, Scherer GW (2001). Computer simulation of mechanical structure–property relationship of aerogels. J. Non-Cryst. Solids.

[20] Schaedler TA (2011). Ultralight Metallic Microlattices. Science.

[21] Zheng X (2014). Ultralight, ultrastiff mechanical metamaterials. Science.

[22] Meza LR, Das S, Greer JR (2014). Strong, lightweight, and recoverable three-dimensional ceramic nanolattices. Science.

[23] Lee J-H, Wang L, Kooi S, Boyce MC, Thomas EL (2010). Enhanced Energy Dissipation in Periodic Epoxy Nanoframes. Nano Lett..

[24] Jang D, Meza LR, Greer F, Greer JR (2013). Fabrication and deformation of three-dimensional hollow ceramic nanostructures. Nat. Mater..

[25] Bauer J, Hengsbach S, Tesari I, Schwaiger R, Kraft O (2014). High-strength cellular ceramic composites with 3D microarchitecture. Proc. Natl. Acad. Sci..

[26] Schaedler TA (2014). Designing Metallic Microlattices for Energy Absorber Applications. Adv. Eng. Mater..

[27] Zheng X (2016). Multiscale metallic metamaterials. Nat. Mater..

[28] Lee J-H, Wang L, Boyce MC, Thomas EL (2012). Periodic Bicontinuous Composites for High Specific Energy Absorption. Nano Lett..

[29] Zhang Q (2009). Energy-Absorbing Hybrid Composites Based on Alternate Carbon-Nanotube and Inorganic Layers. Adv. Mater..

[30] Wang L, Boyce MC, Wen C-Y, Thomas EL (2009). Plastic Dissipation Mechanisms in Periodic Microframe-Structured Polymers. Adv. Funct. Mater..

[31] Greaves GN, Greer AL, Lakes RS, Rouxel T (2011). Poisson’s ratio and modern materials. Nat. Mater..

[32] Bagal A (2015). Multifunctional nano-accordion structures for stretchable transparent conductors. Mater. Horiz..

[33] Bauer J, Schroer A, Schwaiger R, Kraft O (2016). Approaching theoretical strength in glassy carbon nanolattices. Nat. Mater..

[34] Meza LR (2015). Resilient 3D hierarchical architected metamaterials. Proc. Natl. Acad. Sci..

[35] Zhang XA (2015). Ordered 3D Thin-Shell Nanolattice Materials with Near-Unity Refractive Indices. Adv. Funct. Mater..

[36] Chang C-H (2011). From Two-Dimensional Colloidal Self-Assembly to Three-Dimensional Nanolithography. Nano Lett..

[37] Jiang P, McFarland MJ (2004). Large-Scale Fabrication of Wafer-Size Colloidal Crystals, Macroporous Polymers and Nanocomposites by Spin-Coating. J. Am. Chem. Soc..

[38] Min J-H, Zhang XA, Chang C-H (2016). Designing unit cell in three-dimensional periodic nanostructures using colloidal lithography. Opt. Express.

[39] Sherby OD, Wadsworth J (2001). Ancient blacksmiths, the Iron Age, Damascus steels, and modern metallurgy. J. Mater. Process. Technol..

[40] Oliver Wc, Pharr Gm (1992). An improved technique for determining hardness and elastic modulus using load and displacement sensing indentation experiments. J. Mater. Res..

[41] Yokokawa R (2004). Mechanical properties of aerogel-like thin films used for MEMS. J. Micromechanics Microengineering.

[42] Gong B (2011). Sequential Vapor Infiltration of Metal Oxides into Sacrificial Polyester Fibers: Shape Replication and Controlled Porosity of Microporous/Mesoporous Oxide Monoliths. Chem. Mater..

[43] Spagnola JC (2010). Surface and sub-surface reactions during low temperature aluminium oxide atomic layer deposition on fiber-forming polymers. J. Mater. Chem..

[44] Jur JS (2010). Temperature-Dependent Subsurface Growth during Atomic Layer Deposition on Polypropylene and Cellulose Fibers. Langmuir.

