# Supplementary material for: Large-Area Nanolattice Film with Enhanced Modulus, Hardness, and Energy Dissipation
Source: Sci Rep. 2017 Aug 22;7:9145. doi: 10.1038/s41598-017-09521-6 (PMC5567370; doi:10.1038/s41598-017-09521-6)
Supplement: Supplementary file 1 — Supplementary Information [file 41598_2017_9521_MOESM1_ESM.pdf]

Large-Area Nanolattice Film with Enhanced Modulus, Hardness, and Energy Dissipation

Abhijeet Bagal, [1] *Xu A. Zhang,* [1] *Rahnuma Shahrin,* [2] *Erinn C. Dandley,* [3] *Junjie Zhao,* [3] *Felipe R. Poblete,* [1] *Christopher J. Oldham,* [3] *Yong Zhu,* [1] *Gregory N. Parsons,* [3] *Christopher Bobko,* [2] *and Chih-Hao Chang* [*, 1]

Supplementary Information A: Fabrication process for 3D nanolattice samples

The proposed fabrication process is shown in Figure S1, which begins with patterning a periodic 3D photoresist template using colloidal phase lithography.[1] This photoresist template can then be conformably coated with thin films of various material compositions using ALD. In this work, Al_2O_3 and ZnO were selected to fabricate the lightweight, ultra-strong nanolattice material. A photoresist template conformally coated with 40 nm Al_2O_3 is shown in Figure 1b. The polymer template can then be removed by using a combination of solvent dissolution, and/or thermal treatment, leaving behind a free-standing periodic thin-shell structure as shown in Figure 1c. This process results in nanometer-level precision in designing nanolattice period and unit-cell geometry through nanolithography, while the shell thickness can be controlled by ALD with sub-nanometer accuracy. The porosity of the fabricated nanolattice film depends on the remaining shell thickness, which can control the material's modulus, hardness, and energy dissipation.

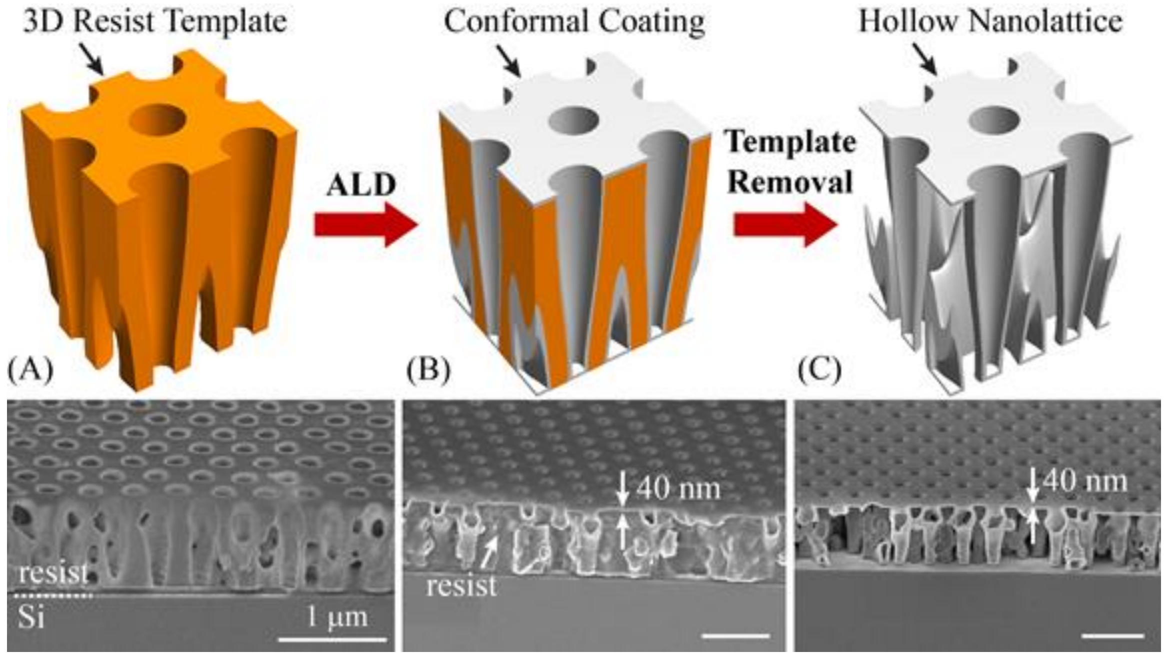

Figure S1. Fabrication process of ordered 3D nanolattice thin-film. **a.** A 3D photoresist template was fabricated using colloidal phase lithography. SEM image shows ordered 3D photoresist structure (D = 500 nm, λ = 325 nm). **b.** Photoresist template was conformally coated using atomic layer deposition technique. SEM image shows 40 nm Al_2O_3 conformally coated on photoresist template. **c.** Photoresist template removal to achieve hollow shellular 3D nanolattice film. SEM shows a free-standing 3D nanolattice with 40 nm shell thickness.

Supplementary Information B: Cross-sectional images of nanolattice samples

All the nanolattice samples tested for mechanical properties have a free-standing thin-shell structure. Figure S2 shows the cross-sectional micrographs of remaining nanolattice samples. All the samples have architectural arrangement consisting of primary hollow tubular columns surrounded by secondary thin-shell structures. The secondary shell structure helps to further enhance the robustness of the nanolattice.

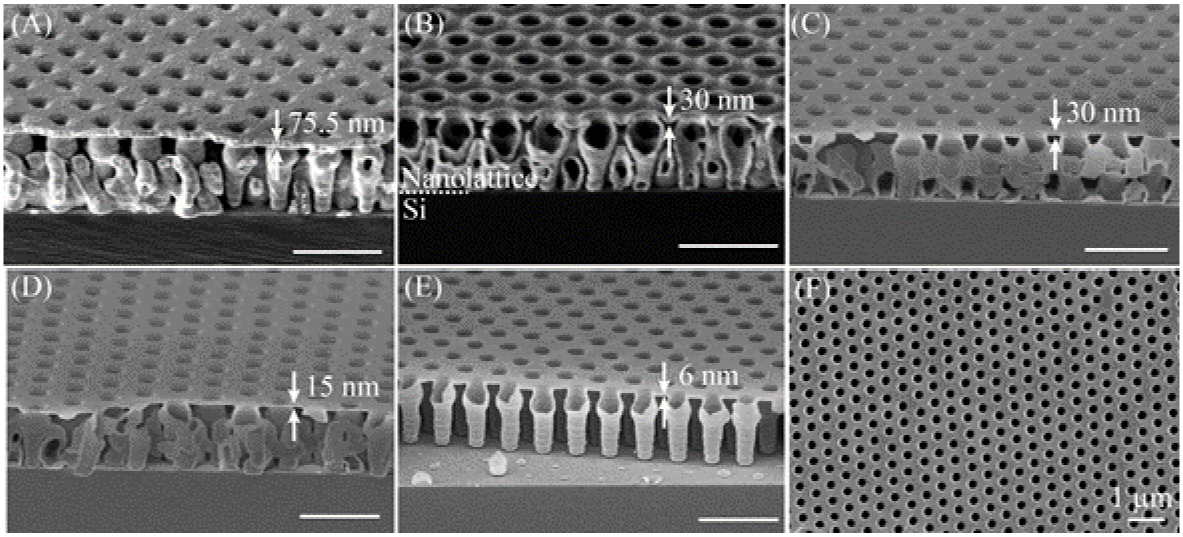

Figure S2. Scanning electron micrographs of samples used for mechanical testing. **a-b.** Cross-sectional image of ZnO nanolattices with thicknesses 45 nm and 75.5 nm respectively. **c-d.** Cross-sectional image of Al_2O_3 nanolattices with thicknesses 30 nm, 20 nm, 15 nm, and 6 nm respectively. **f.** Top-view SEM of 15 nm Al_2O_3 nanolattice

ZnO nanolattices with shell thickness smaller than 30 nm were difficult to fabricate, as the structure collapses during the template removal process. Figure S3 shows the top view and cross-sectional SEM image of 10 nm ZnO nanolattice after removing the resist template. The porous morphology is due to the polycrystalline nature of ALD ZnO.

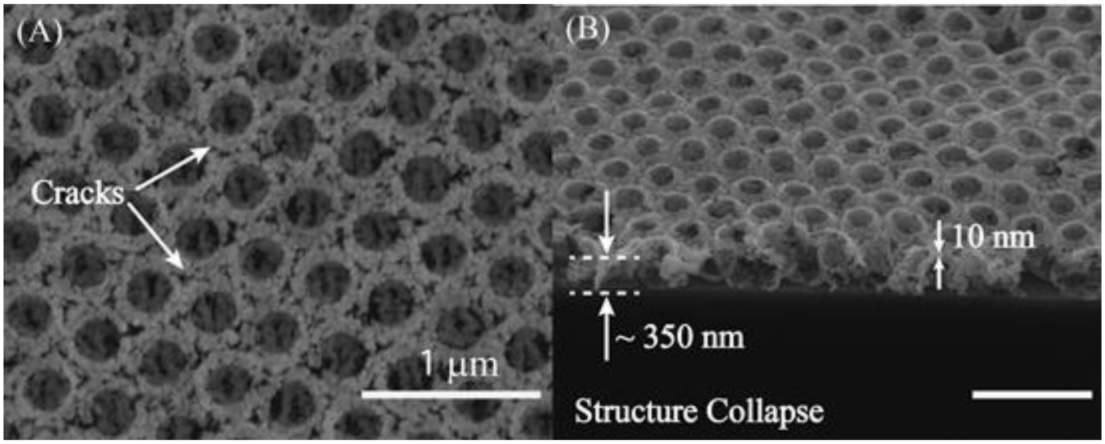

Figure S3. 10 nm ZnO nanolattice. **a.** Top view SEM showing cracks in the nanolattice after removing resist template. **b.** Structure collapses readily due to cracks.

Supplementary Information C: Nanoindentation of nanolattice material

The schematic in Figure S4a shows load P applied through a spherical diamond indenter on a uniform nanolattice film of thickness $t = 1$ µm on silicon substrates. The spherical diamond indenter has a radius of 10 µm and covers a few unit cells of nanolattice film during indentation. The nanolattice film was tested for mechanical properties using the cyclic incremental loading approach. This approach involves selecting a maximum load to be applied on a nanolattice sample, which then will be divided over 10 loading-unloading cycles with exponential increment in load. The maximum load and incremental load were kept constant for a particular sample while indenting at multiple locations. Details of maximum load and incremental loads for various nanolattice samples are listed in Table S1. During each unloading sub-cycle, the nanoindenter was unloaded to 5% of the load applied in that cycle. This ensured that the load was applied at the exact same location during all subsequent incremental cycles by maintaining continuous contact between indenter and sample surface. This also helped in avoiding possible errors that may arise during initial imperfect contact if the indenter was fully unloaded.

Table S1: Loads used in cyclic incremental loading of nanolattice films

Shell thickness (nm)		Incremental loads (μN)									
		1	2	3	4	5	6	7	8	9	10
Al_2O_3	40	50	90	157	349	635	963	1366	1837	2383	3000
	30	50	70	137	267	437	654	920	1231	1592	2000
	20	50	60	79	108	159	227	295	383	486	600
	15	50	54	68	90	120	158	206	262	328	400
	10	25	27	33	44	60	79	103	131	164	200
	6	25	26	28	33	40	49	48	71	84	100
	4	10	16	21	27	32	38	43	49	55	60
ZnO	95	50	90	157	349	635	963	1366	1837	2383	3000
	75.5	50	68	120	222	341	499	698	930	1199	1500
	58	50	62	83	122	168	251	331	438	566	700
	45	50	64	93	143	219	313	428	567	722	900
	30	50	57	73	100	139	190	251	323	399	500

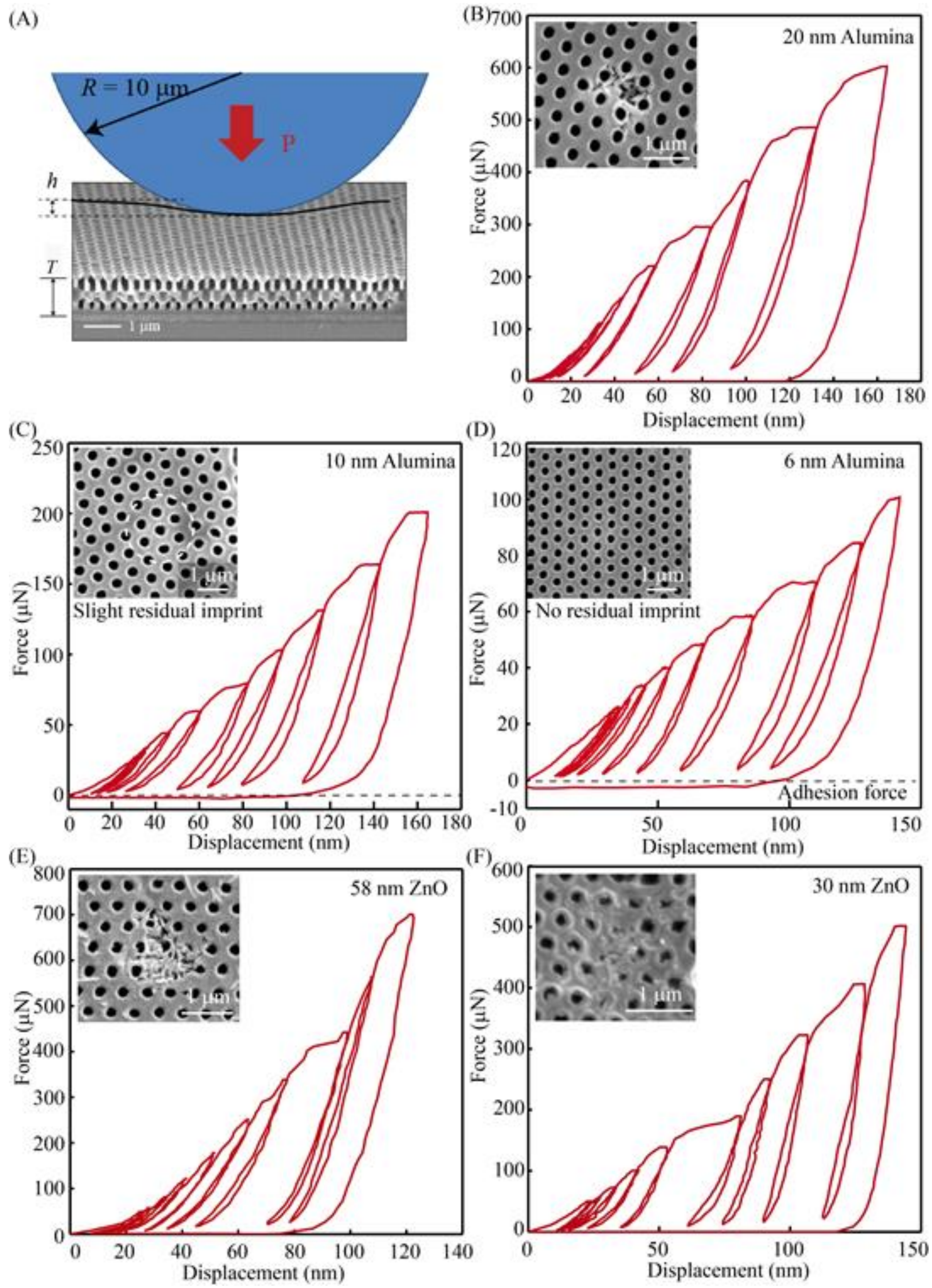

Figure S4. Load-displacement curves. **a.** Schematic of nanoindentation mechanism. **b-d.** load-displacement curves for 20 nm, 10 nm and 6 nm Al_2O_3 nanolattices respectively. **e-f.** load-displacement curves for 58 nm and 30 nm ZnO nanolattices respectively.

The load-displacement curve for 20 nm Al_2O_3 nanolattice shown in Figure S4b has smooth loading-unloading and sharper 'pop-in', indicating brittle failure. This was confirmed by post-indent top-view SEM which showed that the top planer layer has cracks around holes resulting from brittle fracture. Load-displacement curves for 10 nm and 6 nm Al_2O_3 nanolattice are shown in Figure S4c and d respectively. Both the nanolattices have shell thickness smaller than the critical thickness which causes these samples to fail by buckling of the primary tubular column during indentation. The serrated load-displacement curve and gradual 'pop-in' indicate ductile-like behavior resulting from shell buckling of the tubular columns. The non-zero value of adhesion force for both these samples indicates that they recovered completely when the sample was unloaded. Figure S4e and f plot load-displacement curve for 30 nm and 58 nm ZnO nanolattices. These curves show similar behavior as thickerAl_2O_3 nanolattice samples (t >10 nm) with the occurrence of 'pop-in' after a few incremental loading cycles, indicating structure failure resulting from cracking of top planer layer. The maximum indentation load applied varied from 60 µN to 3000 µN for the samples tested.

The nanolattices have few imperfections arising from particle assembly defects during the self-assembly step. To ensure the data collected during nanoindentation was from defect-free regions, post indent SEM images of all the samples were taken. Figure S5 shows the load-displacement curve for a 45 nm ZnO nanolattice when the indent was made on a defect. Due to the presence of the defect, the structure collapsed during loading. Due to structure collapse, a dramatic increase in displacement was recorded at lower loads. Inset shows SEM with indentation residue on a defect. Only the nanoindentation data collected from defect-free regions were considered for calculating mechanical properties of the nanolattice. All the samples studied in this

work were tested at 30 different locations. Out of these tests, about 15-25% were rejected due to the indents made on grain boundaries or point defects.

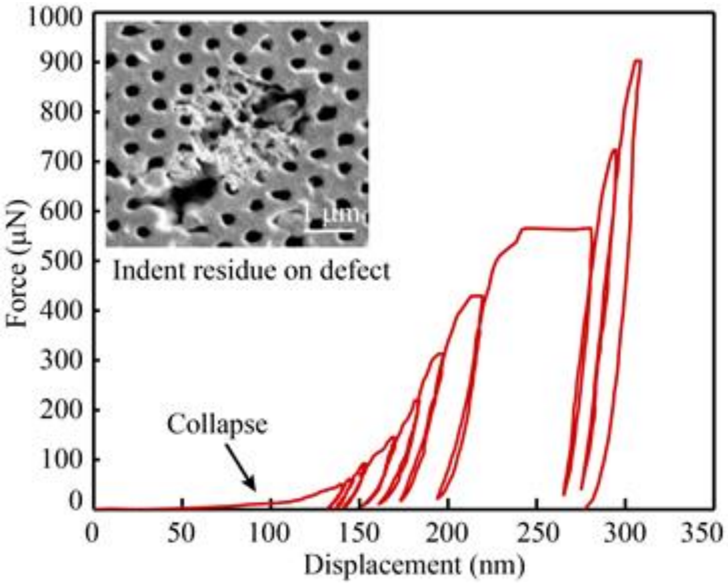

Figure S5. Load-displacement curve for 45 nm ZnO nanolattice on a defect.

Supplementary Information D: Calculation of mechanical properties of nanolattice

Indentation modulus calculation:

The modulus of the nanolattice samples can be determined from the slope (S) of the unloading curve of load-displacement response of the sample, as shown in Figure 2a. The indentation modulus is related to this slope through the contact area (A) during indentation and indenter geometry based correction factor (β);

$$Indentation\ Modulus = \frac{S\sqrt{\pi}}{2\beta\sqrt{A}}$$

The contact area for spherical indenter depends in indenter radius (R) and contact depth (h_c) measured during indentation.

$$A = 2\pi R h_c$$

The indentation modulus was calculated at different locations for each nanolattice sample. These values were later averaged to get indentation modulus value for each sample. Values for all the samples tested are listed in Table S3.

Specific energy dissipation calculation:

Energy dissipated by the nanolattice film was the plastic work done on the structure during compressive loading. To calculate the specific energy dissipation by nanolattice film, the plastic work done in all the loading cycles before 'pop-in' was added together to get the cumulative plastic work. This cumulative plastic work was then divided by the total volume (V) displaced by the spherical nanoindenter during loading to calculate energy dissipation per unit volume (J/mm^3). To accurately analyze the energy absorbing properties of the nanolattice material, the total failure volume zone within the lattice during nanoindentation was evaluated by finite element modeling

of the nanolattice. This volume is important in estimating the specific energy dissipation, since the inelastic energy of the nanolattice material from the force-displacement curve is attributed to irreversible damage within the lattice film elements.

Finite element modeling (FEM):

Three-dimensional (3D) finite element analysis (FEA) of the indentation of Al_2O_3 and ZnO nanostructures were carried out in Abaqus 6.14. The nanolattice structure was modeled as a plate supported by thin-shell hollow pillars with average radius of 125 nm in a square lattice with spacing of 500 nm. The 3D FEA model is illustrated in Figure S6. A quarter of the nanolattice structure was modeled in view of the 4-fold symmetry of the structure.

For both Al_2O_3 and ZnO nanostructures, isotropic linear elastic behaviors and 20-node quadratic brick with reduced integration elements (C30D20R) were used. The mechanical

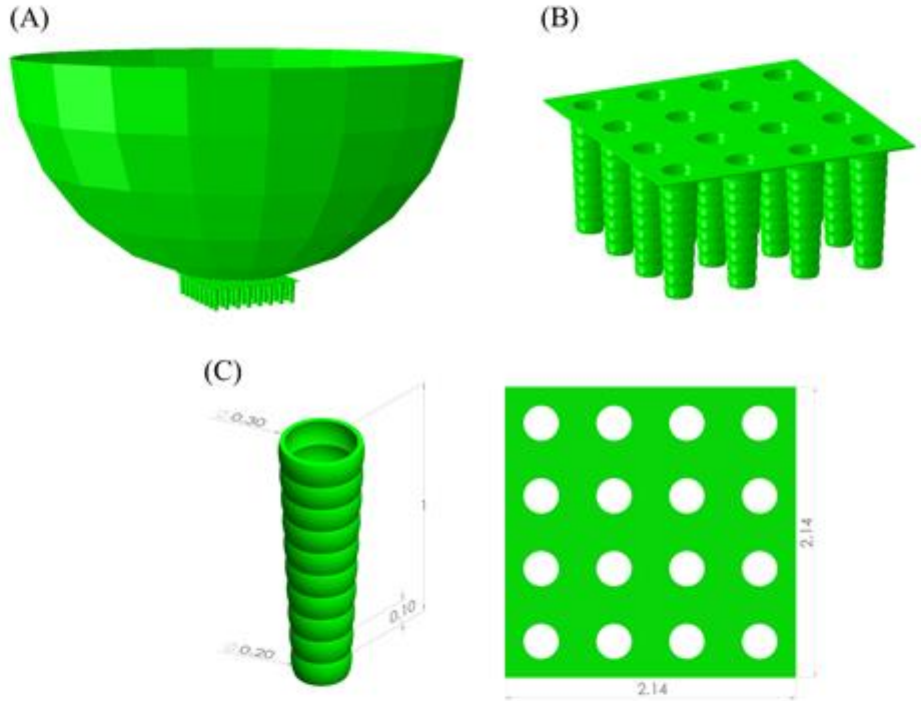

Figure S6. Nanolattice indentation modeled in FEA. **a.** Indenter tip positioned over the entire nanolattice. **b.** Actual nanolattice model analyzed considering a quarter symmetry. **c.** Basic structure dimensions. The dimensions are in μm.

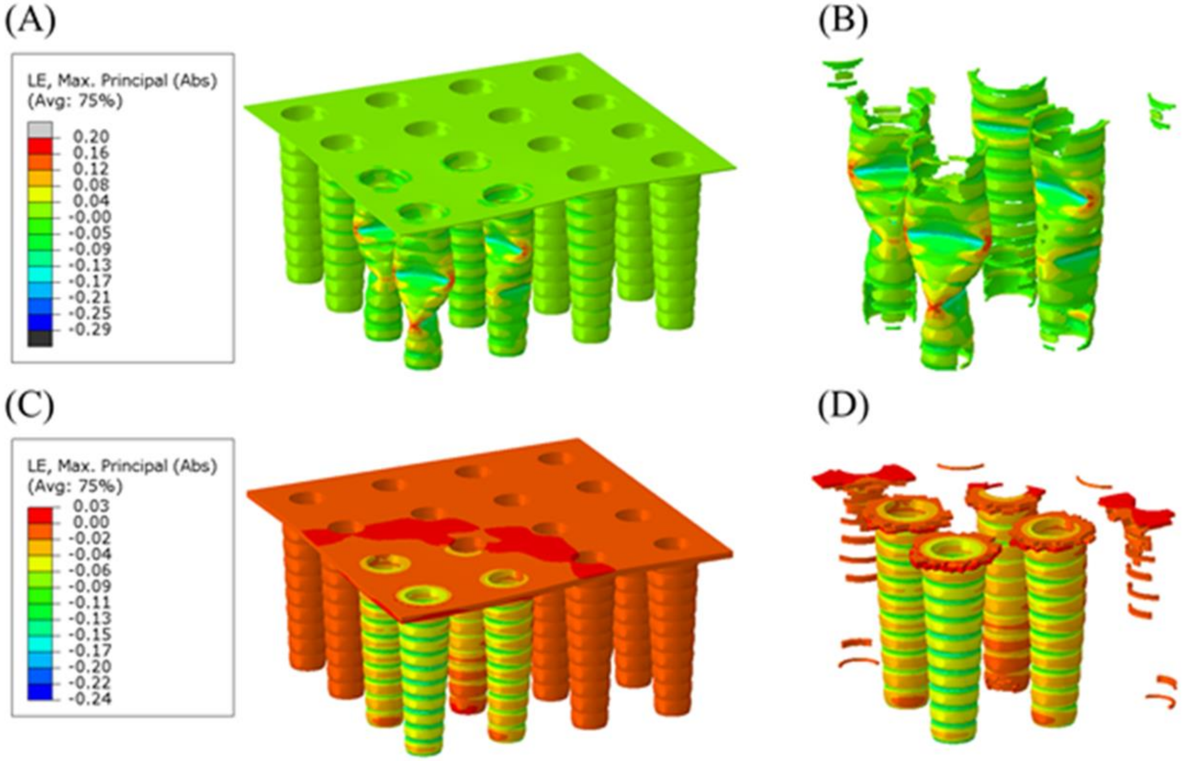

Figure S7. Maximum principal strain for the 10 nm and 40 nm thick Al_2O_3 structure. **a.** Strain distribution across the entire 10 nm thick structure. **b.** Part of the structure above the critical strain value for the 10 nm thick structure. **c.** Strain distribution across the entire 40 nm thick structure. **d.** Part of the structure above the critical strain value for the 40 nm thick structure.

properties of the individual shell elements are based on experimental values reported in literature. For Al_2O_3, a Young's modulus of 180 GPa and a Poisson's ratio of 0.24 were assigned[2] whereas for ZnO, a Young's modulus of 146 GPa and a Poisson's ratio of 0.30.[3] The shell thicknesses of both structures were varied to reflect the fabricated structures. The indenter tip was modeled as an analytical rigid structure with a diameter of 20 μm. In each simulation, distribution of the maximum principal strain was recorded. To calculate the energy dissipation, the volume in which the maximum principal strain is equal or above the critical strain value was used.

Representative results from the FEA simulations are shown for 10 and 40 nm thick Al_2O_3 structure are shown in Figure S7. Figure S7(a) and (c) depict maximum principal strain contours at the maximum indentation depth of 80.23 and 82.32 nm for the 10 and 40 nm samples, respectively. The movie of the FEA simulation has been included as Supplementary Information

(Movie A and B). To obtain the volume, the nanolattice model was subjected to the same indentation depth used in the experiments. Figure S7(b) and (d) depict the volume where the local strain exceeds the critical strain of 2 and 1.05% for the 10 and 40 nm samples, respectively. The total volume was multiple by a factor of 4 to calculate the total failure volume. Buckling was observed for Al_2O_3 structures with thickness below 30 nm. For these cases, in order to avoid convergence issues, artificial damping was added to the model. It was assured that the ratio between the artificial damping energy and the strain energy of the model was no more than 5%. A table summarizing the FEA simulation results for all samples are shown below in Table S2.

Table S2: FEA Calculation of failure volume zone of nanolattice films

Shell thickness (nm)		Indentation Depth (nm)	FEA Volume (μm^3)	Critical Compressive Strain (%)	Critical Tensile Strain (%)
Al_2O_3	**4**	91.78	0.028	2	2.41
	**6**	86.78	0.05	2	2.41
	**10**	80.23	0.10	2	1.62
	**15**	62.97	0.12	2	1.40
	**20**	97.29	0.28	2	1.19
	**30**	83.61	0.38	1.03	1.16
	**40**	82.32	0.50	1.05	0.95
ZnO	**30**	72.59	0.28	2	2
	**45**	96.61	0.56	2	2
	**58**	52.96	0.32	2	2
	**75.5**	83.73	0.71	2	2
	**95**	74.73	0.74	2	2

The specific energy dissipation for a nanolattice was calculated by dividing the energy dissipation per volume by samples density. For 4 nm Al_2O_3 nanolattice, the averaged value of contact depth was 91.78 nm at 50 μN load, for which the total deformed volume from FEA was calculated to be $0.28*10^8$ nm^3. The load-displacement curves for all the nanolattice samples show hysteresis during cyclic loading of the samples. This hysteresis over-predicts the plastic work done during each cycle, and are corrected by removing the hysteresis area when calculating the energy dissipation calculation for all the nanolattice samples. Figure S8 shows the representative hysteresis loops before pop-in for the load-displacement curve of 4 nm Al_2O_3 nanolattice shown in Figure 2c. The hysteresis error was in the range of 6.01 – 17.41% for all the samples. The corrected averaged cumulative plastic work for 4 nm nanolattice was 1.16 pJ. Therefore the energy dissipated per volume by 4 nm Al_2O_3 nanolattice is;

$$Energy\ dissipation\ per\ volume = \frac{W_p}{V} = 41.43 * 10^3\ kJ/m^3$$

The specific energy dissipated by 4 nm Al_2O_3 nanolattice was calculated by dividing the energy dissipation per volume by its density (127.6 kg/m^3).

$$Specific\ energy\ dissipation\ = \frac{41.43 * 10^3}{127.6} = 325.5\ kJ/kg$$

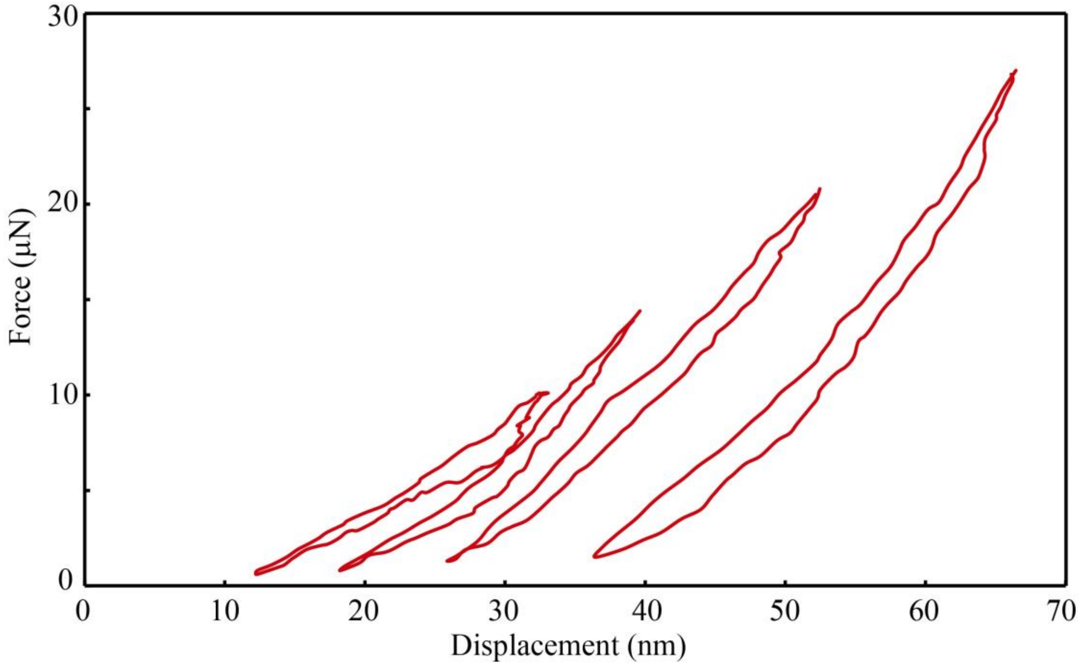

Figure S8. Hysteresis loops for 4 nm Al_2O_3 nanolattice

Hardness calculation:

The hardness of nanolattice samples is calculated by dividing the load by indenter contact area at the pop-in event. Hardness is calculated at pop-in event as it signifies first mechanical failure of the nanolattice with fully developed plastic zone.

$$Hardness = \frac{Load}{Contact\ area\ (A)}$$

Elastic recovery calculation:

The elastic recovery of Al_2O_3 nanolattices was calculated by considering the difference between the maximum indentation depth during the load-displacement curve and the depth of residual imprint. These values were calculated at all the defect-free locations for each sample and averaged to get elastic recovery for individual nanolattice samples. The errors bars plotted in Figure 3d are the standard deviation for the elastic recovery of each sample.

Table S3: Mechanical properties of nanolattice

Shell thickness (nm)		Relative density (%)	Density (kg/m^3)	Indentation modulus (Gpa)	Specific energy dissipation (kJ/kg)	Total energy absorbed (pJ)	Hardness (Mpa)
Al_2O_3	**4**	4.12	127.6	1.19 ± 0.26	325.5	1.16	7.85 ± 0.99
	**6**	6.18	188.5	2.27 ± 0.46	307.24	3.28	12.06 ± 1.21
	**10**	9.89	316.1	3.39 ± 0.90	168.45	6.08	24.12 ± 5.31
	**15**	16.74	464.0	6.98 ± 1.85	109.33	6.83	56.04 ± 8.53
	**20**	21.86	609.3	8.05 ± 1.83	95.39	17.33	65.73 ± 9.87
	**30**	30.73	876.96	11.56 ± 1.30	85.27	30.43	138.26 ± 20.74
	**40**	40.97	1133.9	16.06 ± 3.53	67.2	40.54	-
**ZnO**	**30**	34.36	1927.8	7.75 ± 1.57	12.07	7.11	46.07 ± 7.16
	**45**	42.60	2389.86	10.47 ± 2.27	11.98	16.77	59.41 ± 8.12
	**58**	51.99	2916.639	12.05 ± 2.88	7.54	7.74	113.78 ± 13.58
	**75.5**	62.85	3525.885	14.7 ± 3.79	9.65	27.67	127.86 ± 14.35
	**95**	72.78	4082.958	19.03 ± 5.12	10.82	36.97	181.78 ± 16.62

Supplementary Information E: Failure mode analysis

To analyze the failure mode, the geometry of a primary tubular column in the nanolattice material was simplified as a hollow cylindrical column. During ALD, the outer radius R of this column remains constant and the thickness t of the layer increases towards the center. From SEM images, the averaged outer radius was estimated to be about 125 nm. The aspect ratio of tubular columns in nanolattice was about 4:1, which prevents the Euler buckling failure mode. So the nanolattice failed either by fracture of top planer layer or by shell buckling of tubular column. The equation for stress required to fracture a thin plate is:

$$\sigma_{fracture} = \frac{K_c}{\sqrt{\pi t}}$$

Where, K_c is the fracture toughness of the ALD material. The fracture toughness of 1.89 MPa*m$^{0.5}$ and 1.4 MPa*m$^{0.5}$ were used for Al_2O_3 and ZnO, respectively.[4,5] The stress required for shell buckling of hollow cylindrical column is given by:

$$\sigma_{shell} = \frac{E}{\sqrt{3(1-v^2)}}\left(\frac{t}{R}\right)$$

where E is the Young's modulus of ALD material and v is Poisson's ratio. The moduli of 165 GPa and 129 GPa, and Poisson's ratios of 0.24 and 0.349 were used for Al_2O_3 and ZnO, respectively.[6,7]

In Figure S9, the fracture strength (black solid line) and critical strength for shell buckling (red solid line) are plotted as a function of thickness of ALD layer. The failure mode for the nanolattice depends on the shell thickness. The thickness at which these two curves cross each other is the critical thickness (t_{cr}) and it marks the change in failure mode mechanism. For thicker shells, the nanolattice fails by fracturing of the top planer layer as indicated by the solid black line.

And for thinner shells the nanolattice fails by buckling of the cylindrical column. The nanolattices used in this work have lattice height (h) of 1 μm. For this configuration, the critical thickness for failure mode change-over was 11.5 nm for Al_2O_3 nanolattices. Micro-scale lattices with larger outer radius (R) of cylindrical column fail by buckling at higher thicknesses (solid blue line), resulting in lower stiffness. The sub-micron period of the nanolattices fabricated in this work has resulted in tubular structures with a smaller outer radius, which helps in strengthening the structure and attaining higher stiffness. The nanolattice can be strengthened further by lowering the outer radius of the hollow cylindrical columns and by having higher packing density. But such close packed arrangement will result in denser nanolattice material.

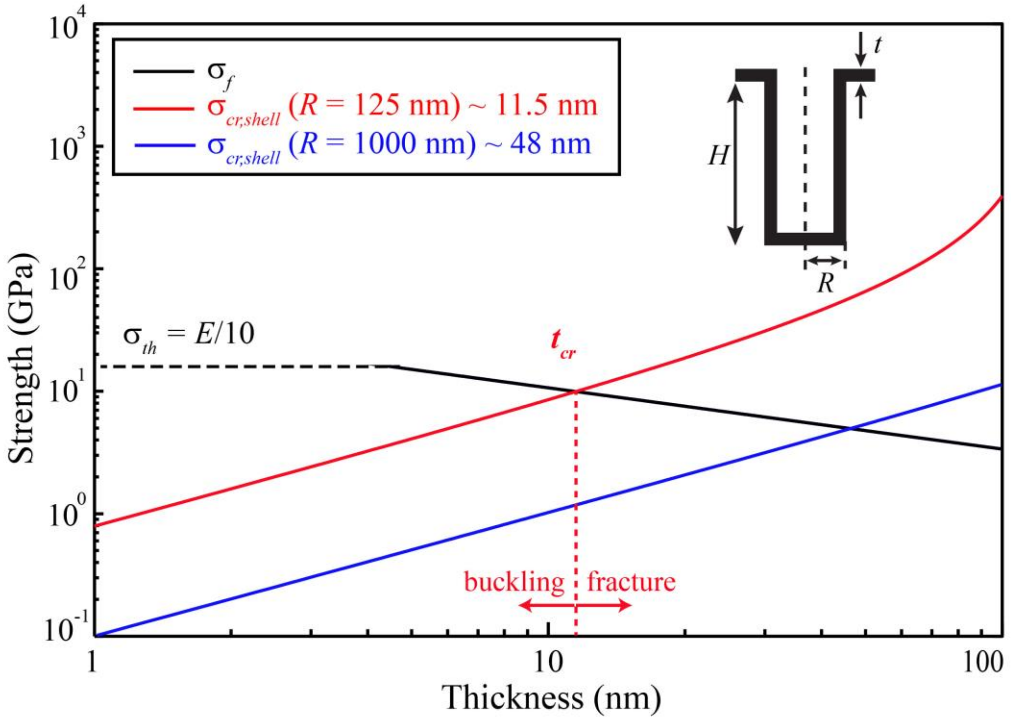

Figure S9. Failure mode analysis of Al_2O_3 nanolattice.

The critical thickness for failure mode changeover between sheet fracturing and tubular column buckling for ZnO nanolattice was 10.9 nm. For ZnO nanolattice samples tested in this

work, the shell thickness ranged from 30 nm – 95 nm, so all the samples fail by fracturing of the top planer layer around holes. Figure S10 shows the failure mode analysis for ZnO nanolattice.

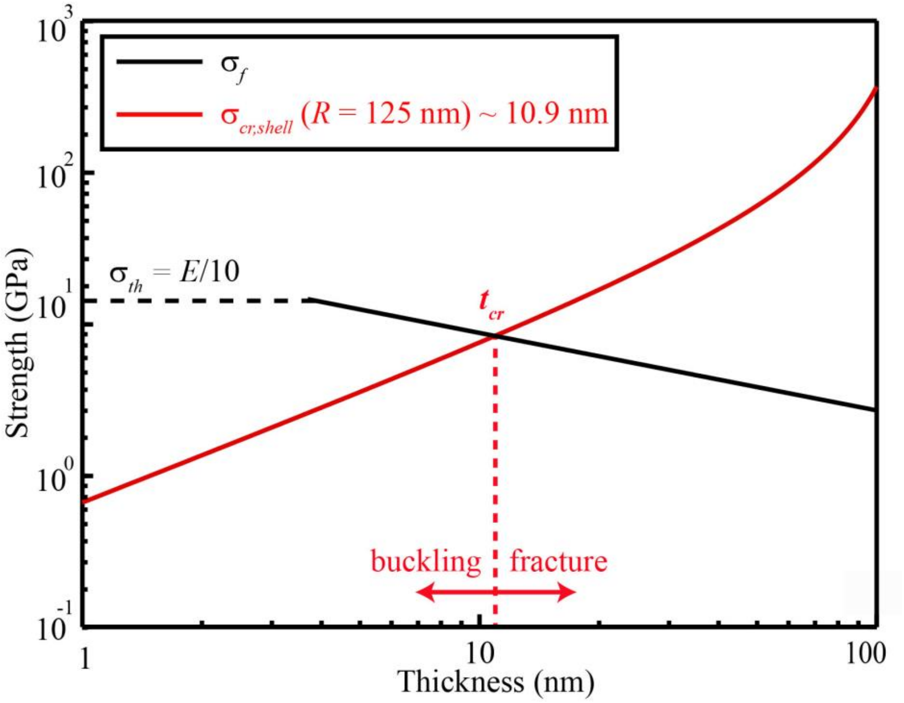

Figure S10. Failure mode analysis of ZnO nanolattice.

Supplementary Information F: Recoverability analysis of Al_2O_3 nanolattice

The post-indent top-view SEM images of Al_2O_3 nanolattices with shell thickness lower than the critical thickness do not have the indentation residue like the thick-shell nanolattice samples. The failure mode analysis indicates that thinner lattices fail by buckling of the tubular column during compressive loading. The top planer layer gets elastically stretched during loading and remains undamaged unlike thicker nanolattices. When these thinner nanolattices were unloaded, the buckled tubular columns elastically recovered to regain their shape. This shape recovery was also facilitated by the elastically deformed top-planer layer regaining its original shape. This recovery can be observed from the load-displacement curve of thinner Al_2O_3 nanolattices ($t \leq$ 10 nm), which show a negative force after complete unloading of the indenter. This negative force is the adhesion force resulting from the Van der Waal's attraction between spherical diamond indenter and the recovering nanolattice film. This non-zero adhesion force confirms that the nanolattice was in contact with the diamond indenter even when the tip has retracted, since the film the recovered to its original height. To verify this negative force is due to contact, the Van der Waal's forces between the tip and the nanolattice film can be calculated. The surface energy of Al_2O_3 and diamond are 2.6 J/m^2 and 5.24 J/m^2, respectively.[8] The total work of adhesion at the interface is given by:

$$\gamma = \gamma_{Al_2O_3} + \gamma_{Diamond} - 2\sqrt{\gamma_{Al_2O_3} * \gamma_{Diamond}}$$

The adhesion force acting between the nanolattice and the spherical diamond indenter is related to the contact radius a through the indenter radius R and indentation depth h, which is given by:

$$a = \sqrt{Rh}$$

The top layer of nanolattice in contact with the diamond indenter has hexagonal symmetry of holes, which reduces the actual contact area between the nanolattice and indenter. For thinner nanolattices, this area was about 0.43 times the total area. By assuming this contact area to be circular, the contact radius was calculated to be about 235 nm for 4 nm Al_2O_3 nanolattice. Under external loading conditions, the contact between the nanolattice and indenter remains Hertzian.[9] Therefore, the force of adhesion is given by:

$$P_A = \sqrt{8\pi a^3 2\gamma E^*}$$

The adhesion force calculated by this method was 18.8 μN, which is about five times the averaged adhesion force obtained from load-displacement curves. From the load-displacement curves, the averaged values for adhesion force are 3.07 μN, 3.08 μN and 2.66 μN for 4 nm, 6 nm and 10 nm alumina nanolattices, respectively. The lower value of adhesion force in experimental results could be due to the imperfect contact between diamond indenter and the nanolattice surface. The presence of the Van der Waal's adhesion force demonstartes the tip and film maintain contact, indicating that the films have recovered to the original height.

The negative adhesion force was also observed for thicker nanolattices ($t \geq$ 10 nm) during unloading. However, unlike the thinner nanolattices, the force goes to zero before the indenter is completely unloaded. This demonstrates that the tip loses contact with the film during unloading, indicating nanolattices with $t > t_{cr}$ do not show complete recovery due to brittle fracture. Figure S8 shows the adhesion force for 40 nm Al_2O_3 nanolattice, which is representative of the indentation curve for thicker shells. The averaged values for adhesion force are 1.77 μN, 1.41 μN, 0.57 μN, and 1.92 μN for 15 nm, 20 nm, 30 nm, and 40 nm alumina nanolattices, respectively. Note these values are comparable to the thinner shell samples, which is expected given the tip and nanolattice

materials are the same. The adhesion for the thicker nanolattice films may be smaller due to non-conformal contact of the more rigid film.

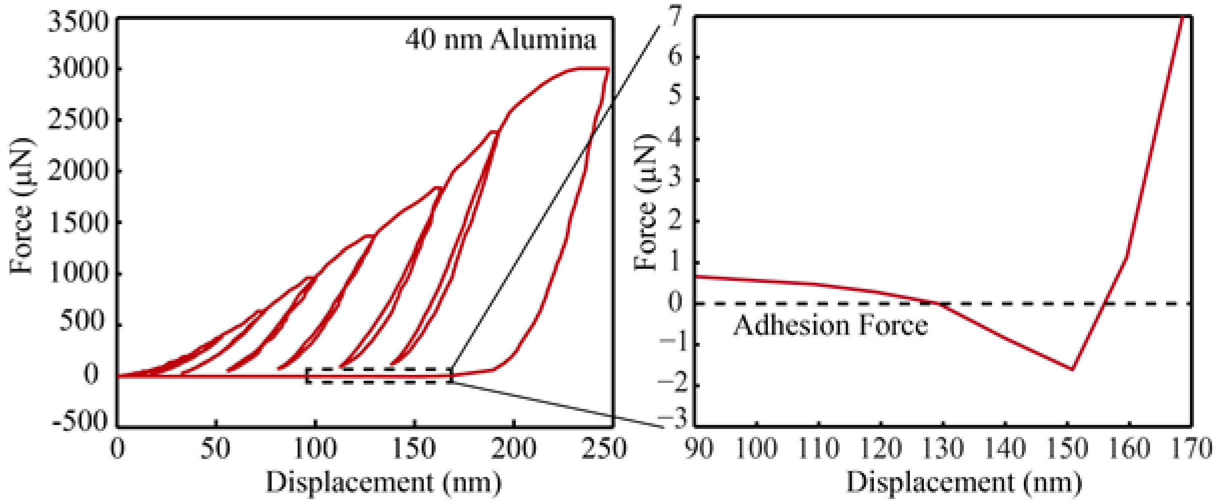

Figure S11. Adhesion force for 40 nm Al_2O_3 nanolattice.

References:

[1] C.-H. Chang, L. Tian, W. R. Hesse, H. Gao, H. J. Choi, J.-G. Kim, M. Siddiqui, G. Barbastathis, *Nano Lett.* **2011**, *11*, 2533.

[2] *J. Appl. Phys.* **2011**, *109*, 084305.

[3] K. Tapily, D. Gu, H. Baumgart, G. Namkoong, D. Stegall, A. A. Elmustafa, *Semicond. Sci. Technol.* **2011**, *26*, 115005.

[4] D. C. Miller, R. R. Foster, Y. Zhang, S.-H. Jen, J. A. Bertrand, Z. Lu, D. Seghete, J. L. O'Patchen, R. Yang, Y.-C. Lee, S. M. George, M. L. Dunn, *J. Appl. Phys.* **2009**, *105*, 093527.

[5] R. Riedel, I.-W. Chen, *Ceramics Science and Technology, Materials and Properties*; John Wiley & Sons, 2011.

[6] M. Berdova, T. Ylitalo, I. Kassamakov, J. Heino, P. T. Törmä, L. Kilpi, H. Ronkainen, J. Koskinen, E. Hæggström, S. Franssila, *Acta Mater.* **2014**, *66*, 370.

[7] Y. Gao, Z. L. Wang, *Nano Lett.* **2009**, *9*, 1103.

[8] S. P. Adiga, P. Zapol, L. A. Curtiss, *Phys. Rev. B* **2006**, *74*, 064204.

[9] A. C. Fischer-Cripps, In *Nanoindentation*; Mechanical Engineering Series; Springer New York, 2011; pp. 235–276.
